# A flexible whole-genome microarray for transcriptomics in three-spine stickleback (*Gasterosteus aculeatus*)

**DOI:** 10.1186/1471-2164-10-426

**Published:** 2009-09-11

**Authors:** Erica H Leder, Juha Merilä, Craig R Primmer

**Affiliations:** 1 Division of Genetics and Physiology, Department of Biology (Vesilinnantie 5), University of Turku, FI-20014 Turku, Finland; 2 Ecological Genetics Research Unit, Department of Biological and Environmental Sciences, PO Box 65, FI-00014 University of Helsinki, Helsinki, Finland

## Abstract

**Background:**

The use of microarray technology for describing changes in mRNA expression to address ecological and evolutionary questions is becoming increasingly popular. Since three-spine stickleback are an important ecological and evolutionary model-species as well as an emerging model for eco-toxicology, the ability to have a functional and flexible microarray platform for transcriptome studies will greatly enhance the research potential in these areas.

**Results:**

We designed 43,392 unique oligonucleotide probes representing 19,274 genes (93% of the estimated total gene number), and tested the hybridization performance of both DNA and RNA from different populations to determine the efficacy of probe design for transcriptome analysis using the Agilent array platform. The majority of probes were functional as evidenced by the DNA hybridization success, and 30,946 probes (14,615 genes) had a signal that was significantly above background for RNA isolated from liver tissue. Genes identified as being expressed in liver tissue were grouped into functional categories for each of the three Gene Ontology groups: biological process, molecular function, and cellular component. As expected, the highest proportions of functional categories belonged to those associated with metabolic functions: metabolic process, binding, catabolism, and organelles.

**Conclusion:**

The probe and microarray design presented here provides an important step facilitating transcriptomics research for this important research organism by providing a set of over 43,000 probes whose hybridization success and specificity to liver expression has been demonstrated. Probes can easily be added or removed from the current design to tailor the array to specific experiments and additional flexibility lies in the ability to perform either one-color or two-color hybridizations.

## Background

Microarrays and other whole genome methods are increasingly being applied to examine transcription patterns relevant for ecology and evolution in wild populations (e.g[1,2]). One of the obstacles to applying this technology to non-model organisms is sufficient sequence data from which array features can be designed. Because of this, many of the arrays used for non-model organisms have been cDNA arrays spotted from cDNA clones since whole-genome sequence information does not exist (e.g[1,3]). This limits the number of genes to those found in cDNA libraries which may not be representative of the whole genome. Additionally, in many cases heterologous hybridizations are used where one uses an array designed from another closely related species (reviewed in [4]). However, for many study organisms this is not feasible since the species of interest are evolutionarily too distant from species for which a microarray is available.

The evolutionary significance of three-spine stickleback (*Gasterosteus aculeatus*) stems from its well-documented history of parallel episodes of colonization from marine habitats followed by population divergence [5]. Phenotypic divergence has resulted in trophic morphs [6], variation in lateral plate numbers [7], and pelvic reduction [8]. Many of these morphotypes can be associated with selective pressures of a given habitat (e.g. increased armour under high predation risk) [9,10]. The genetic architecture of several adaptive traits (e.g. gill raker number, spine length) has been known for a number of years [11] and yet, despite the efforts to identify genes responsible for phenotypic traits important for adaptive divergence, only two genes have been suggested as playing a role in these processes, *EDA *and *pixt1 *[7,8]. Therefore, tools to study adaptive evolution at the transcriptome level would be of great value for gaining a deeper understanding of the evolution of this organism.

The three-spine stickleback was one of the first ecological and evolutionary model species to have its genome sequenced .

However, the full potential of a whole-transcriptome analysis has yet to be fully realized for this species; despite having a sequenced genome, only one cDNA microarray, based on EST sequences, has been published for use with sticklebacks [12]. This array contains 9,692 clones which is less than half of the estimated gene number in the stickleback genome.

In an effort to develop tools to facilitate transcriptomic studies in three-spine sticklebacks, we used known genes and novel gene predictions from the sequenced genome of three-spine stickleback in Ensembl to create probes for use with the Agilent microarray format. In this array design, 43,654 probes were created representing 19,274 genes which is approximately 93% of the estimated genome (20,787 known, novel, and projected protein coding genes). This array design accounts for differential splicing by creating transcript-specific probes whenever possible. Since these probes are created *in situ *for the Agilent array platform, the actual array design is flexible and can be modified to suit the needs of specific experiments, and user-designed probes can also be added. We demonstrate the utility of the array through examining hybridization success of DNA from 6 individuals from a total of four populations as well as RNA from 17 individuals from a total of three populations.

## Results and Discussion

### Probe design

**Figure 1:**
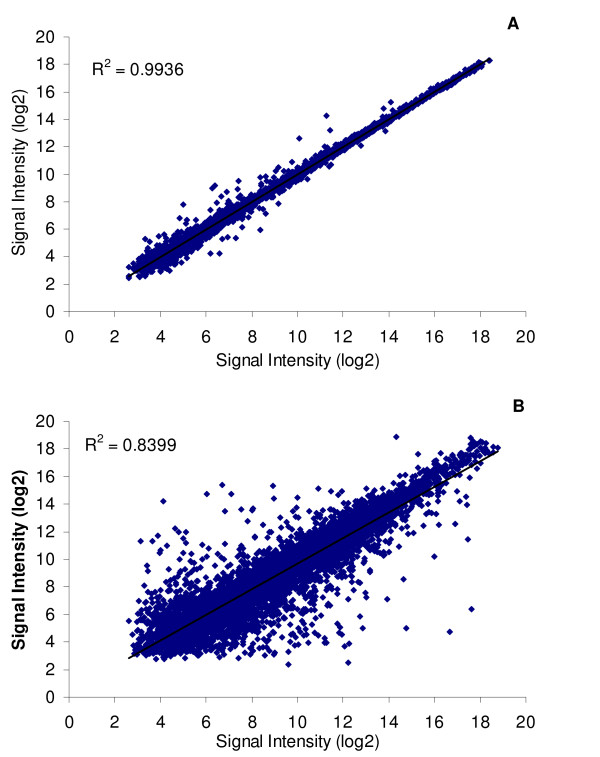
**Correlation of different probes from the same transcript**. Signal intensity (log2) from the median of 17 RNA samples of the probe pairs from the same transcript when the difference of the probes' sequence position is a) within 5 or fewer bases (N = 6,427 transcripts; R^2 ^= 0.994) or b) greater than 5 bases (N = 7,192 transcripts; R^2 ^= 0.840). Of the two probes, the probe on the X-axis was closer to the 3' end of the transcript.

**Figure 2:**
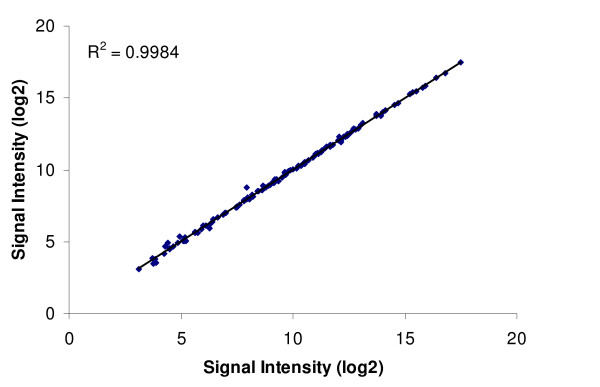
**Correlation of replicated probes**. Signal intensity (log2) from the median of 17 RNA samples of replicated probe pairs (131 pairs; R^2 ^= 0.998).

From 27,723 transcripts, 43,392 unique probes representing 19,274 genes were selected (Additional file 1 - STable 1). Since these probes were designed to be transcript specific, splice variants were represented in the probe design if possible. In addition, since the number of features on the array allowed for more than the number of predicted transcripts, two different probes were designed for each transcript if possible. For 18,569 genes, between 2 and 13 probes were designed and for the remaining 705 genes, only one probe was designed. For 6,427 transcripts the probes were very similar, differing by just 1 to 5 bases in sequence position along the transcript. Despite having almost the same sequence, these probes were retained in the initial design since it was unclear how similar fish from the European lineage would be to those from the sequenced lineage (North American), and base pair differences can impact the binding affinity depending on the location of the mismatch [13]. However for most transcripts, when RNA was hybridized, a difference of a few bases in an optimal binding area did not affect the signal intensity (Figure 1a). When probes were designed further apart along a transcript, the signal intensity was affected although it was still highly correlated (Figure 1b). Due to the *in situ *probe creation process, probes are high quality and the background signal is quite low. However, due to unused space, there were 255 probes that were replicated on the array (7 of these are in triplicate). Of the replicated probes with a significant hybridization, there was almost perfect correlation among these probes (Figure 2). Array design is available at  accession number: A-MEXP-1443.

### DNA hybridizations

**Figure 3:**
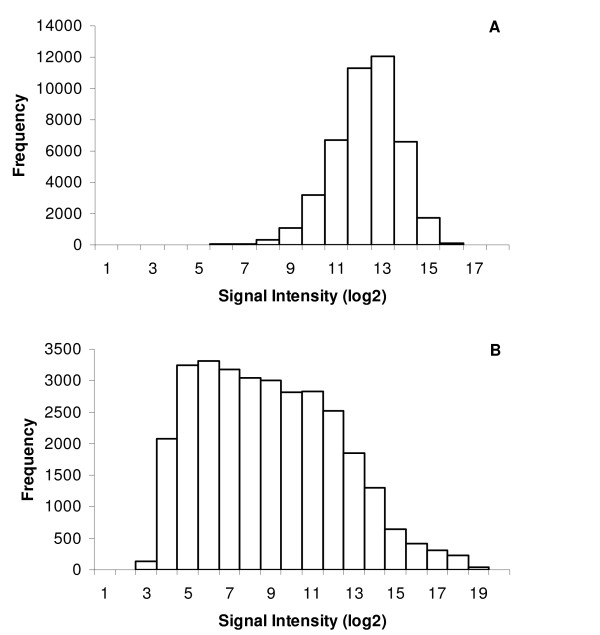
**Hybridization signal intensity**. Frequency distribution of signal intensity of: A) DNA samples hybridized to array (median of 6 individuals) B) RNA samples hybridized to the array (median of 17 individuals).

To determine the overall effectiveness of the probes, DNA was hybridized to the arrays. For six individuals, virtually all probes (43,648 of 43,654) had an intensity value significantly above background in all individuals. Only two probes clearly did not hybridize as the signal from these probes was not significant in all but one individual. These two probes were designed from the same transcript, however two other transcripts from the same gene had significant signal intensities. Five additional probes had 1-3 individuals that were not significantly above background. The hybridization signal intensity was quite high for many probes for the DNA samples (Figure 3a), and above the maximum detectable level for 512 probes. Of these, 352 were saturated in all 3 arrays for the Cy5 channel as Cy5 gets saturated before Cy3. When the 512 saturated probes were removed, the pairwise correlations of signal intensity over all probes for the 6 individuals ranged from 0.95-0.98.

### RNA hybridizations

For liver tissue mRNA, 30,946 out of 43,654 probes (71%) had a signal intensity that was significantly above background for at least 15 out of 17 individuals. These probes represented 14,615 genes (75.8%). A more conservative threshold using probes flagged as "WellAboveBG" was also applied to these data. With this threshold, 25,213 probes (11,871 genes, 61.6%) were considered significantly expressed in liver tissue. The proportion of significantly expressed genes is higher than observed in a study using a similar array system in mice, where 12, 845 (54.5%) of the genes surveyed were considered "transcriptionally active" [14]. However, the lower proportion of genes expressed in the liver tissue of mice can likely be explained by the rigorous selection scheme employed by the authors to identify the "most transcriptionally active" genes. This difference also illustrates the idea that the suitability of certain probes for inclusion in a particular study will depend on the specific experimental question, and despite having a signal higher than the background, some probes may not be appropriate for some experiments due to their relatively low intensity.

The distribution of signal intensity for RNA was much more varied than with DNA and many more features had low signal intensity (Figure 3b). This is expected since the copy number of the majority of genes in DNA should be the same, and the signal intensity of DNA hybridizations is based more on binding affinity and the size of the bound DNA fragments since more label will be incorporated in larger fragments.

### Functional annotation

Three-spine stickleback genes that exhibited a signal well above background (15 of 17 arrays flagged IsWellAboveBG) (11,871 genes) were examined for functional characterization. Genes were initially matched to their putative human orthologs using Biomart from Ensembl . BLAST searches were also used to increase the annotation information. From Biomart, a human UniProt accession number was obtained if possible to be used for functional characterization since the stickleback genes are not sufficiently annotated with regard to function. Approximately 2,959 genes (out of 11,871) which had a significant intensity were not assigned a human UniProt accession number.

Functional categories were assigned using Go Term Mapper  and the human generic GO Slim categories. Genes were placed into the "slimmed" categories for biological process (BP), molecular function (MF), and cellular component (CC). There were 877 unannotated genes of the 8,912 that had UniProt accession numbers.

The largest proportion of genes were involved in metabolism (BP: 54.3%), binding (MF: 73.5%), specifically protein binding (48.2%) and catabolism (MF: 36.7%). Other than cell (85.5%) and intracellular (70.5%), organelles were the most represented category of the cellular component (58.0%). These results are consistent with expectations as the liver plays an important role in metabolism, and these functions occur in organelles, mostly mitochondria.

**Figure 4:**
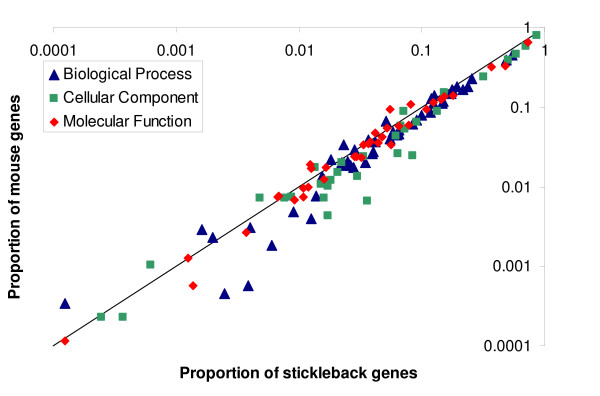
**Comparison of liver tissue mRNA expression in mouse and stickleback**. Comparison of the proportion of genes involved in a specific GO ontology term for mouse and three-spine stickleback. Genes that were significantly expressed in liver tissue were mapped to their respective functional categories with Go Term Mapper. For each GO category in the GO Slim generic subset of terms, the proportion of genes representing that term for mouse was plotted against the proportion of genes representing that term for stickleback. (See Additional file 2 - STable 2 for numerical values and GO terms). The line represents a 1:1 relationship. Axes are on a logarithmic scale.

As further evidence that the mRNA expressed in this experiment are characteristic of liver tissue expression, we compared our results to the transcriptionally active genes from mouse liver tissue [14]. Using Go Term Mapper, the mouse (MGI) generic Go Slim, and the mouse gene symbols from the active genes, we obtained the proportion of genes in each of the Go Slim functional categories for mouse liver expression. From 10,939 genes, 102 had ambiguous IDs and 2,136 were unannotated, leaving 8,701 genes for functional annotation. The proportions of genes in each of the Go Slim categories were highly correlated between stickleback and mouse liver expression (Spearman Rank correlation: Biological Process, Rho = 0.981, P = 7.33E-36; Molecular Function, Rho = 0.984, P = 7.21E-28; Cellular Component, Rho = 0.928, P = 6.25E-14) (Additional file 2 - STable 2, Figure 4).

## Conclusion

The Agilent Gene Expression Array Platform provides an extremely flexible custom array format for creating arrays for expression analysis. Because of the *in situ *probe creation, there is low background which leads to a high degree of reproducibility between array batches. Approximately 43,000 probes, representing 19,274 genes, are now available for use for transcriptomics studies of three-spine stickleback. Probes can easily be added or removed from the current design to tailor the array to specific experiments as not all genes will be expressed in all tissues. Additional flexibility lies in the ability to perform either one-color or two-color hybridizations. Both types of hybridizations were used successfully in the testing of the arrays.

## Methods

### Sampling of the study populations and rearing of the offspring

RNA sampled from fish from three study populations were tested on the arrays: Helsinki (Baltic Sea; 60°10'N, 25°00'E), Lake Pulmanki (Finnish Lapland; 69°58'N, 27°58'E) and Lake Vättern (Sweden; 58°54'N, 14°24'E). Full-sib F1 families were created by crossing parental fish at the sampling sites, and fertilized eggs were transported to the laboratory. Initially, the offspring were maintained with water at 17 ± 1°C and a photoperiod of 18 h light 6 h dark, and six months after hatching the environmental conditions were gradually changed to complete darkness (24 h dark) and 9 ± 1°C to simulate wintering conditions. After five months the environmental conditions were changed back to 18/6h L/D photoperiod, 17 ± 1°C and new crosses were made to obtain F2 offspring from each population. At the time of the experiments the F2 offspring were approximately 20 months old. They were adult fish, although at the time of the experiment, reproductively inactive.

### Experimental sampling

Fish were sampled from the tanks and immediately euthanized with a lethal dose of MS-222 anesthetic. Livers were removed from fish immediately, frozen in liquid nitrogen, and stored at -80°C. Six fish per population were analyzed for RNA, but one array (from the Pulmanki population) was of poor quality and was removed from the analysis. Since fish were not reproductively active, it was impossible to determine sex before the treatment. Subsequent dissection and visualization of reproductive structures revealed that there were 4 females and 13 males in the experiment.

In order to determine the functionality of the array, two representatives from each of the above populations and a fourth population, representing an evolutionarily divergent lineage, was used for testing DNA hybridization to the arrays. The two additional DNA samples are from two individuals from the Pacific Ocean lineage collected from Shiomi River, Japan (43°01'N, 144°50'E). However, one array, containing individuals from Lake Vättern and Lake Pulmanki, was removed from analysis due to poor quality.

### Microarray design

Microarrays were designed using the custom gene expression 4 × 44K platform from Agilent which consists of 4 arrays per slide with 45,220 features, 43,803 of which are user-defined 60-base pair (bp) oligonucleotide probes. It is a newer configuration of that which was previously described [15]. There are 153 negative controls, and 1264 positive controls including spike-in control probes for an external RNA reference. The Agilent control features aid the Feature Extraction software in orienting the grid and applying within array normalization.

Probes were designed in eArray 4.5 (Agilent) using known and novel transcripts from Ensembl Stickleback Assembly Broad S1, database version 42.1b . All known and novel transcripts (as defined by Ensembl) (27,723) were included initially for two separate probe design projects in eArray using the default options. Probes were designed using the Base Composition Methodology option in eArray which is an Agilent methodology which chooses probes based on empirically determined base-composition profiles. The option to choose probes biased towards the 3' end was selected as recommended by Agilent. With this option, the software attempts to design the probe within the first 1000 bases of the transcript. In the first project, two probes for each transcript were designed using the input transcripts as the 'genome' to prevent cross-hybridization. For the second project, all parameters were the same except stickleback EST sequences from NCBI were used as the 'genome'. The two different input 'genomes' were used in the attempt to detect other splicing variants that may not have been recognized in the genome database. The probe sets from the two projects were combined and lower quality probes (as assessed according to parameters in the methodology-rating 3 BC and 4 BC) were removed. Additionally, the majority of duplicate probes resulting from the two different design jobs were removed.

There are 43,654 stickleback probes on the microarray, and of these, 43,392 are unique as some duplicated probes were retained. The majority of probes were within 500 bases of the 3' end (83.5%), 14.7% were between 501 and 1000 bases from the 3' end, and only 1.8% were greater than 1000 bps from the end. Additionally, 149 randomly chosen probes from the Agilent catalogue probe sets were used to fill the array. The initial intention was that these may be used as negative controls; however due to conserved gene sequence, stickleback RNA and DNA hybridized to some of the probes, so they were all removed from the analysis.

### DNA sample preparation and array hybridisation

DNA was extracted from stickleback liver tissue using the Qiagen DNA Easy Kit using RNaseA treatment. DNA was eluted 2 times using 50 μL of water each time. 750 ng of DNA was digested with 5 units of AluI and 5 units of RsaI for 2 hours at 37°C and followed by 20 min at 65°C. The digested DNA was examined on an agarose gel to assess the effectiveness of the digestion.

DNA labelling, hybridizations, and scanning were performed by the Finnish DNA Microarray Centre, which is an Agilent certified service provider. The Agilent protocol for Oligonucleotide Array-Based CGH for Genomic DNA Analysis was performed. Briefly, after digestion, DNA samples were either Cy3 or Cy5 labeled with Agilent's Genomic DNA labelling kit following manufacturer's protocols. After labelling, the DNA concentrations and specific activity was checked using the Nanodrop ND-1000 (NanoDrop Technologies).

The Cy3 labelled sample and the Cy5 labelled sample were hybridized together onto Agilent's Gene Expression 4 × 44K custom array designed for three-spine stickleback at 65°C for 24 hours using Agilent's Oligo aCGH/ChIP-Chip Hybridization Kit. Washes were conducted with Agilent's Oligo aCGH/ChIP-on-Chip Wash Buffer set using Agilent's Stabilization and Drying solution according to the protocol. Arrays were scanned with Agilent Technologies Scanner, model G2505B. Spot intensities and other quality control features were extracted with Agilent's Feature Extraction Software version 9.5.3.1.

### RNA sample preparation and array hybridisation

Total RNA was isolated by means of Tri Reagent (Sigma) using the manufacturer's protocol. RNA was treated with DNase (Promega), 2.5 units for 1-2 μg RNA, and re-isolated using Tri Reagent. RNA concentration was quantified using a Nanodrop ND-1000, and RNA quality was assessed using an Experion Automated Electrophoresis System (Bio-Rad).

RNA labelling, hybridizations, and scanning were performed by the Finnish DNA Microarray Centre. Briefly, total RNA (400 ng) was amplified and Cy3-labeled with Agilent's Low RNA Input Linear Amplification Kit PLUS, One Color (Agilent) along with Agilent's One-Color RNA Spike-in Kit following the manufacturer's protocols. After the labelling, the cRNA was examined with the Nanodrop ND-1000 and the Experion Automated Electrophoresis System cRNA to assess the concentration and quality of the labelling. Each sample (1.65 μg) was hybridized to the custom designed stickleback array at 65°C overnight (17 h) using Agilent's GE Hybridization Kit. Washes were conducted as recommended by the manufacturer using Agilent's Gene Expression Wash Pack without any stabilization or drying solution. Arrays were scanned with Agilent Technologies Scanner, model G2505B. Spot intensities and other quality control features were extracted with Agilent's Feature Extraction Software version 9.5.3.1. Array experiments are available at ArrayExpress with the accession numbers E-MEXP-2304 and E-MEXP-2309.

### Data analysis

Array quality was assessed through the use of Agilent control features as well as spike-in controls (Agilent 1-Color Spike-in Kit for RNA experiment). Due to poor hybridization, one array from the RNA experiment and one array from the DNA experiment were removed from further analysis. Processed signals from the Feature Extraction Software (v 9.5.3.1) were used for the analysis. Agilent's Feature Extraction software automatically normalizes within arrays, subtracts the background, and flags any outlier spots, either due to saturation or non-uniformity. Further details can be obtained from the Feature Extraction user guide . The software also determines the features which should be kept in the analysis by flagging features that are significantly above background as determined by a two-sided t-test. Features which were flagged as being positive and significant (IsPosAndSignif) were retained as long as that feature was positive and significant in at least 15 of the 17 arrays. Likewise, a more rigorous threshold was applied by using the flag IsWellAboveBG, which first determines if the feature is significant (IsPosAndSignif) and then determines if the background-subtracted signal is greater than 2.6 times the background standard deviation for that feature (approximates a 99% CI). Arrays were normalized using quantile normalization [16] from the Limma package in R/Bioconductor [17] in order to adjust the scale of intensities across arrays.

## Authors' contributions

EL designed the probes and the microarray, conducted the laboratory work (with the exception of RNA labelling and array hybridizations), performed the statistical analysis, and drafted the manuscript. JM and CP supervised the research and assisted with the manuscript preparation. All authors read and approved the final manuscript.

## Supplementary Material

Additional file 1 **STable 1 - Probe information and gene annotation**. Information about the probes including: probe name (Agilent), probe sequence, transcript and gene Ensembl identifiers, flags to determine feature significance, RNA median intensity and interquartile range and DNA median intensity (of 6 individuals). For the flag information for IsPosAndSignif and WellAboveBG, "1" means that the feature met the flag criteria in 15 out of 17 arrays. Click here for file

Additional file 2 **STable 2**. Comparison of the functional categories of liver tissue mRNA expression in mouse and stickleback. Click here for file
