# Supplementary material for: A flexible whole-genome microarray for transcriptomics in three-spine stickleback (Gasterosteus aculeatus)
Source: BMC Genomics. 2009 Sep 11;10:426. doi: 10.1186/1471-2164-10-426 (PMC2754499; doi:10.1186/1471-2164-10-426)
Supplement: Additional file 2 — STable 2. Comparison of the functional categories of liver tissue mRNA expression in mouse and stickleback. [file 1471-2164-10-426-S2.doc]

| GO Term description |  | stickleback  n=8035 | | mouse  n=8701 | |
| --- | --- | --- | --- | --- | --- |
| **Biological Process** | GO accession | Gene counts | Proportion | Gene counts | Proportion |
| metabolic process | GO:0008152 | 4361 | 0.543 | 3900 | 0.448 |
| primary metabolic process | GO:0044238 | 3888 | 0.484 | 3386 | 0.389 |
| secondary metabolic process | GO:0019748 | 16 | 0.002 | 20 | 0.002 |
| DNA metabolic process | GO:0006259 | 325 | 0.040 | 242 | 0.028 |
| protein metabolic process | GO:0019538 | 1726 | 0.215 | 1435 | 0.165 |
| lipid metabolic process | GO:0006629 | 467 | 0.058 | 421 | 0.048 |
| nucleobase, nucleoside, nucleotide and nucleic acid metabolic process | GO:0006139 | 1908 | 0.237 | 1590 | 0.183 |
| cellular amino acid and derivative metabolic process | GO:0006519 | 211 | 0.026 | 159 | 0.018 |
| carbohydrate metabolic process | GO:0005975 | 315 | 0.039 | 228 | 0.026 |
| generation of precursor metabolites and energy | GO:0006091 | 225 | 0.028 | 152 | 0.017 |
| biosynthetic process | GO:0009058 | 1205 | 0.150 | 1023 | 0.118 |
| catabolic process | GO:0009056 | 947 | 0.118 | 751 | 0.086 |
| behavior | GO:0007610 | 146 | 0.018 | 193 | 0.022 |
| cell communication | GO:0007154 | 1554 | 0.193 | 1592 | 0.183 |
| cell cycle | GO:0007049 | 523 | 0.065 | 396 | 0.046 |
| cell death | GO:0008219 | 523 | 0.065 | 443 | 0.051 |
| cell differentiation | GO:0030154 | 961 | 0.120 | 1102 | 0.127 |
| cell growth | GO:0016049 | 102 | 0.013 | 34 | 0.004 |
| cell proliferation | GO:0008283 | 439 | 0.055 | 342 | 0.039 |
| cell recognition | GO:0008037 | 13 | 0.002 | 25 | 0.003 |
| cell-cell signaling | GO:0007267 | 180 | 0.022 | 177 | 0.020 |
| cellular homeostasis | GO:0019725 | 189 | 0.024 | 162 | 0.019 |
| cellular component organization | GO:0016043 | 1184 | 0.147 | 962 | 0.111 |
| cytoplasm organization | GO:0007028 | 1 | 0.000 | 3 | 0.000 |
| mitochondrion organization | GO:0007005 | 73 | 0.009 | 42 | 0.005 |
| cytoskeleton organization | GO:0007010 | 202 | 0.025 | 182 | 0.021 |
| organelle organization | GO:0006996 | 672 | 0.084 | 536 | 0.062 |
| death | GO:0016265 | 525 | 0.065 | 446 | 0.051 |
| growth | GO:0040007 | 182 | 0.023 | 163 | 0.019 |
| anatomical structure morphogenesis | GO:0009653 | 415 | 0.052 | 579 | 0.067 |
| multicellular organismal development | GO:0007275 | 1015 | 0.126 | 1226 | 0.141 |
| embryonic development | GO:0009790 | 186 | 0.023 | 296 | 0.034 |
| symbiosis, encompassing mutualism through parasitism | GO:0044403 | 20 | 0.002 | 4 | 0.000 |
| protein modification process | GO:0006464 | 803 | 0.100 | 683 | 0.078 |
| regulation of biological process | GO:0050789 | 2052 | 0.255 | 1969 | 0.226 |
| regulation of gene expression, epigenetic | GO:0040029 | 32 | 0.004 | 27 | 0.003 |
| signal transduction | GO:0007165 | 1418 | 0.176 | 1431 | 0.164 |
| response to abiotic stimulus | GO:0009628 | 129 | 0.016 | 107 | 0.012 |
| response to biotic stimulus | GO:0009607 | 124 | 0.015 | 118 | 0.014 |
| response to endogenous stimulus | GO:0009719 | 110 | 0.014 | 66 | 0.008 |
| response to external stimulus | GO:0009605 | 336 | 0.042 | 316 | 0.036 |
| response to stress | GO:0006950 | 731 | 0.091 | 593 | 0.068 |
| transcription | GO:0006350 | 997 | 0.124 | 976 | 0.112 |
| translation | GO:0006412 | 281 | 0.035 | 177 | 0.020 |
| transport | GO:0006810 | 1397 | 0.174 | 1293 | 0.149 |
| ion transport | GO:0006811 | 297 | 0.037 | 336 | 0.039 |
| protein transport | GO:0015031 | 507 | 0.063 | 393 | 0.045 |
| respiratory electron transport chain | GO:0022904 | 48 | 0.006 | 16 | 0.002 |
| reproduction | GO:0000003 | 230 | 0.029 | 250 | 0.029 |
| viral reproduction | GO:0016032 | 31 | 0.004 | 5 | 0.001 |
|  |  |  |  |  |  |
| **Molecular Function** |  |  |  |  |  |
| Binding | GO:0005488 | 5908 | 0.735 | 5596 | 0.643 |
| DNA binding | GO:0003677 | 876 | 0.109 | 816 | 0.094 |
| RNA binding | GO:0003723 | 448 | 0.056 | 290 | 0.033 |
| Nucleotide binding | GO:0000166 | 1219 | 0.152 | 1197 | 0.138 |
| Nucleic acid binding | GO:0003676 | 1434 | 0.178 | 1208 | 0.139 |
| Translation factor activity, nucleic acid binding | GO:0008135 | 74 | 0.009 | 60 | 0.007 |
| Chromatin binding | GO:0003682 | 55 | 0.007 | 66 | 0.008 |
| Lipid binding | GO:0008289 | 235 | 0.029 | 205 | 0.024 |
| Protein binding | GO:0005515 | 3871 | 0.482 | 2881 | 0.331 |
| Receptor binding | GO:0005102 | 294 | 0.037 | 303 | 0.035 |
| Carbohydrate binding | GO:0030246 | 100 | 0.012 | 167 | 0.019 |
| Cytoskeletal protein binding | GO:0008092 | 226 | 0.028 | 207 | 0.024 |
| Actin binding | GO:0003779 | 134 | 0.017 | 152 | 0.017 |
| Calcium ion binding | GO:0005509 | 335 | 0.042 | 408 | 0.047 |
| Oxygen binding | GO:0019825 | 11 | 0.001 | 5 | 0.001 |
| Protein tag | GO:0031386 | 1 | 0.000 | 1 | 0.000 |
| Antioxidant activity | GO:0016209 | 30 | 0.004 | 23 | 0.003 |
| Catalytic activity | GO:0003824 | 2949 | 0.367 | 2770 | 0.318 |
| Electron carrier activity | GO:0009055 | 129 | 0.016 | 109 | 0.013 |
| Ion channel activity | GO:0005216 | 102 | 0.013 | 149 | 0.017 |
| Structural molecule activity | GO:0005198 | 260 | 0.032 | 199 | 0.023 |
| Kinase activity | GO:0016301 | 419 | 0.052 | 476 | 0.055 |
| Motor activity | GO:0003774 | 54 | 0.007 | 65 | 0.007 |
| Nuclease activity | GO:0004518 | 88 | 0.011 | 83 | 0.010 |
| Peptidase activity | GO:0008233 | 270 | 0.034 | 293 | 0.034 |
| Hydrolase activity | GO:0016787 | 1162 | 0.145 | 1100 | 0.126 |
| Transferase activity | GO:0016740 | 991 | 0.123 | 993 | 0.114 |
| Phosphoprotein phosphatase activity | GO:0004721 | 96 | 0.012 | 85 | 0.010 |
| Protein kinase activity | GO:0004672 | 304 | 0.038 | 319 | 0.037 |
| Receptor activity | GO:0004872 | 442 | 0.055 | 814 | 0.094 |
| Signal transducer activity | GO:0004871 | 650 | 0.081 | 952 | 0.109 |
| Transporter activity | GO:0005215 | 525 | 0.065 | 497 | 0.057 |
| Neurotransmitter transporter activity | GO:0005326 | 10 | 0.001 | 11 | 0.001 |
| Transcription factor activity | GO:0003700 | 355 | 0.044 | 311 | 0.036 |
| Transcription regulator activity | GO:0030528 | 633 | 0.079 | 513 | 0.059 |
| Translation regulator activity | GO:0045182 | 87 | 0.011 | 64 | 0.007 |
| Enzyme regulator activity | GO:0030234 | 384 | 0.048 | 368 | 0.042 |
|  |  |  |  |  |  |
| **Cellular Component** |  |  |  |  |  |
| cell | GO:0005623 | 6870 | 0.855 | 6888 | 0.792 |
| cell envelope | GO:0030313 | 2 | 0.000 | 2 | 0.000 |
| intracellular | GO:0005622 | 5666 | 0.705 | 5007 | 0.575 |
| extracellular space | GO:0005615 | 271 | 0.034 | 207 | 0.024 |
| extracellular region | GO:0005576 | 577 | 0.072 | 777 | 0.089 |
| organelle | GO:0043226 | 4664 | 0.580 | 4003 | 0.460 |
| mitochondrion | GO:0005739 | 731 | 0.091 | 557 | 0.064 |
| cytoplasmic membrane-bounded vesicle | GO:0016023 | 243 | 0.030 | 116 | 0.013 |
| Golgi apparatus | GO:0005794 | 493 | 0.061 | 377 | 0.043 |
| endoplasmic reticulum | GO:0005783 | 539 | 0.067 | 498 | 0.057 |
| endosome | GO:0005768 | 166 | 0.021 | 131 | 0.015 |
| external encapsulating structure | GO:0030312 | 3 | 0.000 | 2 | 0.000 |
| lipid particle | GO:0005811 | 5 | 0.001 | 9 | 0.001 |
| lysosome | GO:0005764 | 122 | 0.015 | 93 | 0.011 |
| ribosome | GO:0005840 | 138 | 0.017 | 89 | 0.010 |
| vacuole | GO:0005773 | 146 | 0.018 | 105 | 0.012 |
| cytoskeleton | GO:0005856 | 585 | 0.073 | 464 | 0.053 |
| cytosol | GO:0005829 | 678 | 0.084 | 216 | 0.025 |
| cytoplasm | GO:0005737 | 4075 | 0.507 | 3405 | 0.391 |
| chromosome | GO:0005694 | 181 | 0.023 | 173 | 0.020 |
| cilium | GO:0005929 | 39 | 0.005 | 62 | 0.007 |
| microtubule organizing center | GO:0005815 | 139 | 0.017 | 37 | 0.004 |
| nucleolus | GO:0005730 | 289 | 0.036 | 57 | 0.007 |
| nucleoplasm | GO:0005654 | 516 | 0.064 | 224 | 0.026 |
| nuclear chromosome | GO:0000228 | 61 | 0.008 | 63 | 0.007 |
| nucleus | GO:0005634 | 2577 | 0.321 | 2108 | 0.242 |
| nuclear envelope | GO:0005635 | 128 | 0.016 | 62 | 0.007 |
| peroxisome | GO:0005777 | 70 | 0.009 | 64 | 0.007 |
| plasma membrane | GO:0005886 | 1222 | 0.152 | 1321 | 0.152 |
| proteinaceous extracellular matrix | GO:0005578 | 109 | 0.014 | 153 | 0.018 |
| protein complex | GO:0043234 | 1080 | 0.134 | 760 | 0.087 |
